# “My life is under control with these medications”: an interpretative phenomenological analysis of managing chronic pain with opioids

**DOI:** 10.1186/s12891-020-3055-5

**Published:** 2020-01-31

**Authors:** Hanna Ljungvall, Annica Rhodin, Sofia Wagner, Hedvig Zetterberg, Pernilla Åsenlöf

**Affiliations:** 1 Department of Neuroscience, Uppsala University, Box 593, 751 24 Uppsala, Sweden; 2 Department of Surgical Sciences, Uppsala University, Uppsala, Sweden

**Keywords:** Chronic pain, Opioids, Qualitative, Interpretative phenomenological analysis

## Abstract

**Background:**

The use of opioids to relieve chronic pain has increased during the last decades, but experiences of chronic opioid therapy (COT) (> 90 days) point at risks and loss of beneficial effects. Still, some patients report benefits from opioid medication, such as being able to stay at work. Guidelines for opioid use in chronic pain do not consider the individual experience of COT, including benefits and risks, making the first person perspective an important scientific component to explore. The aim of this study was to investigate the lived experience of managing chronic pain with opioids in a sample who have severe chronic pain but are able to manage their pain sufficiently to remain at work.

**Methods:**

We used a qualitative research design: interpretative phenomenological analysis. Ten individuals with chronic pain and opioid therapy were purposively sampled in Swedish tertiary care.

**Results:**

Three super-ordinate themes emerged from the analyses: *Without opioids, the pain becomes the boss*; *Opioids as a salvation and a curse*, and *Acknowledgement of the pain and acceptance of opioid therapy enables transition to a novel self*. The participants used opioids to regain control over their pain, thus reclaiming their wanted life and self, and sense of control over one’s life-world. Using opioids to manage pain was not unproblematic and some of the participants had experienced a downward spiral of escalating pain and uncontrollable opioid use, and stigmatisation.

**Conclusions:**

All participants emphasised the importance of control, regarding both pain and opioid use. To accomplish this, trust between participants and health care providers was essential for satisfactory treatment. Regardless of the potential sociocultural benefits of staying at work, participants had experiences of balancing positive and negative effects of opioid therapy, similar to what previous qualitative research has found.

Measurable improvement of function and quality of life, may justify the long-term use of opioids in some cases. However, monitoring of adverse events should be mandatory. This requires close cooperation and a trusting relationship between the patients and their health care provider.

## Background

**Fig. 1:**
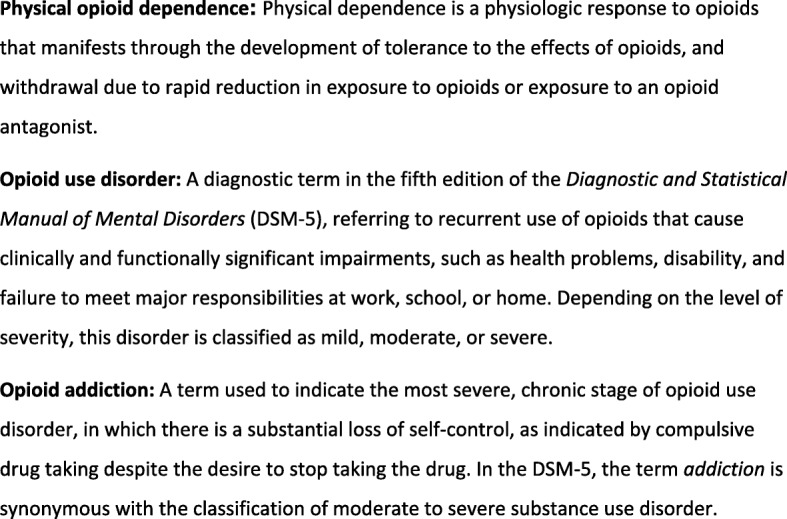
Definitions of physical opioid dependence, opioid use disorder, and opioid addiction [10]

Chronic pain is a major and complex health problem affecting approximately 10–20% of the adult population globally [1, 2]. Biological, psychosocial, and behavioral manifestations of chronic pain are probably inseparable and overlapping [3]. Therefore, management of chronic pain requires an interdisciplinary approach where pharmacological interventions, e.g. opioid therapy, could be part of the treatment [4]. The increasing use of opioids to relieve chronic pain has been apparent in the last few decades [5]. Opioids have been successfully used for acute and cancer pain, and a beneficial effect has been expected for chronic pain [6–8]. Prescription of opioids has therefore skyrocketed in most countries in Europe as well as in the U.S. [7, 9]. However, in the last few years, the problem of opioid dependence, opioid use disorder, and its most severe form, opioid addiction, has been recognised. Definitions of dependence, opioid use disorder and addiction are presented in Fig. 1 [10–12].

Of particular note is the opioid crisis in the U.S., where drug overdoses involving opioids resulted in more than 400,000 deaths during the last two decades as a consequence of this opioid epidemic [13, 14]. Further, the evidence regarding chronic opioid therapy (COT) (opioid therapy > 90 days) for chronic pain [6, 15, 16] is increasingly highlighting the risks, e.g., opioid hyperalgesia, opioid use disorder, and loss of beneficial effects over time [7, 17–25].

Guidelines for opioid use in chronic pain cannot always help in the identification of an appropriate treatment for an individual patient, creating a need for tailored opioid and non-opioid treatment alternatives. Therefore, the first person perspective on managing chronic pain with opioid therapy is essential [7].

Few studies, where most come from North America, have explored the lived experience of managing chronic pain with COT among individuals treated in specialised pain care. Recurrent themes in these studies are stigma related to opioid use, finding a balance between pain and opioid use, and, being questioned by family and health care professionals [26–28]. Vallerand and colleagues [26] found that patients described life before COT as characterised by desperation and loss of function, while life with COT was characterised as balancing between living a secret life, fear of losing the opioids, and thankfulness for a life regained. Brooks et al. [28] found that most of the negative aspects related to opioids were socioculturally induced rather than caused by the drug itself.

This study is part of a project examining the benefits and risks with COT, where ability to work is hypothesised to be a major potential benefit. Here, work is considered as an important sociocultural context where employment is a contributor to social status, well-being and individual identity [29]. There are to the best of our knowledge no studies on managing chronic pain with opioids in a sample being homogeneous in this respect. Therefore, the aim was to explore the lived experience of managing chronic pain with opioids in a sample of individuals with severe chronic pain and the sociocultural benefits of being employed.

## Methods

### Philosophical and theoretical foundations of interpretative phenomenological analysis

In the health sciences, phenomenology has been used in qualitative research for thorough understanding of the lived experience of pain.

Interpretative phenomenological analysis (IPA) is a qualitative research method that examines how people make sense of major life experiences and offers an accessible approach to phenomenological research, intended to give an immersed account of the individual experience [30]. Its theoretical framework is mainly based on phenomenology, hermeneutics, and idiography [31], which makes it possible to explore the ambiguous and elusive nature of the pain phenomenon, especially persistent pain, involving complex bio-psychosocial interactions [32]. Further, it allows for a detailed account of each participant’s experience, both within and between the different accounts.

### Participants and settings

Participants were purposively sampled from a pain clinic and an addiction clinic at a university hospital in Sweden. The clinics are tertiary care units with patients from all over Sweden, offering multimodal, interdisciplinary pain rehabilitation, consultation visits, and pharmacological therapy. The clinics offer both inpatient and outpatient care. The pain clinic treats about 2000 patients per year, and about 170 of these are on COT. The addiction clinic provides treatment for substance use disorders. Patients with chronic pain are referred to the clinic for opioid agonist therapy, where their opioids are switched to methadone or buprenorphine, to induce better pain relief and reduce opioid tolerance, withdrawal symptoms, and cravings. Patients recruited from the addiction clinic were attending an outpatient program that treats about 150 patients with chronic pain and problematic opioid use without current illicit drug use.

Eligible for participation were individuals 18–65 years of age with chronic pain and COT, currently in employment or work rehabilitation, and with work experience. Being a full-time student was considered equivalent to current employment. We aimed to recruit a reasonably homogenous sample of participants, where all had experiences of chronic pain, COT, and work. Thus, we could examine similarities and differences within the sample in some detail as regards COT and chronic pain [31]. A sample size of about 10 individuals was considered to be sufficient, since we aimed for a detailed account of individual experiences, rather than thematic saturation, in accordance with IPA methodology [31].

### Procedures

Following approval from the regional ethical review board in Uppsala (Number 2017/265), information about the study was posted in the clinics’ waiting rooms. It provided contact information to the first author so that interested participants could volunteer for the study. However, a majority of the participants, 9 out of 10, were recruited through physicians who treated patients with chronic pain with opioids. Suitable participants who agreed to be contacted were approached through e-mail or phone by the first author, and received first-hand verbal and written information about the study, including information about the possibility of an observer participating during the interview. Immediately prior to the interviews, consent forms were provided and signed by the participants. Before their interview started, each participant filled out a medical data form providing demographic information and data regarding pain duration and medication. An observer was present in 5 out of 10 interviews and conducted field notes describing the context, flow, and ambience during the interview. If only the interviewer was present, field notes were taken in conjunction with the interview, but not during. Eight out of the 10 interviews took place in the facilities of the addiction clinic, which offered an environment suitable for confidential and sensitive conversations. The facility is open to the public, with a large variety of people coming and going, e.g., patients with various kinds of psychiatric problems (not only substance-related), students, and health care personnel, which makes it a non-stigmatising environment to visit. Upon request from one of the participants, her interview was held in her home. One interview was held at a participant’s workplace, as this was most convenient for her. The interview was conducted in her office with no one present except the participant, the interviewer, and an observer who was a member of the research team.

### Data collection and analysis

Medical data were retrieved from medical records and from the medical data forms completed by the participants prior to the interviews.

**Fig. 2:**
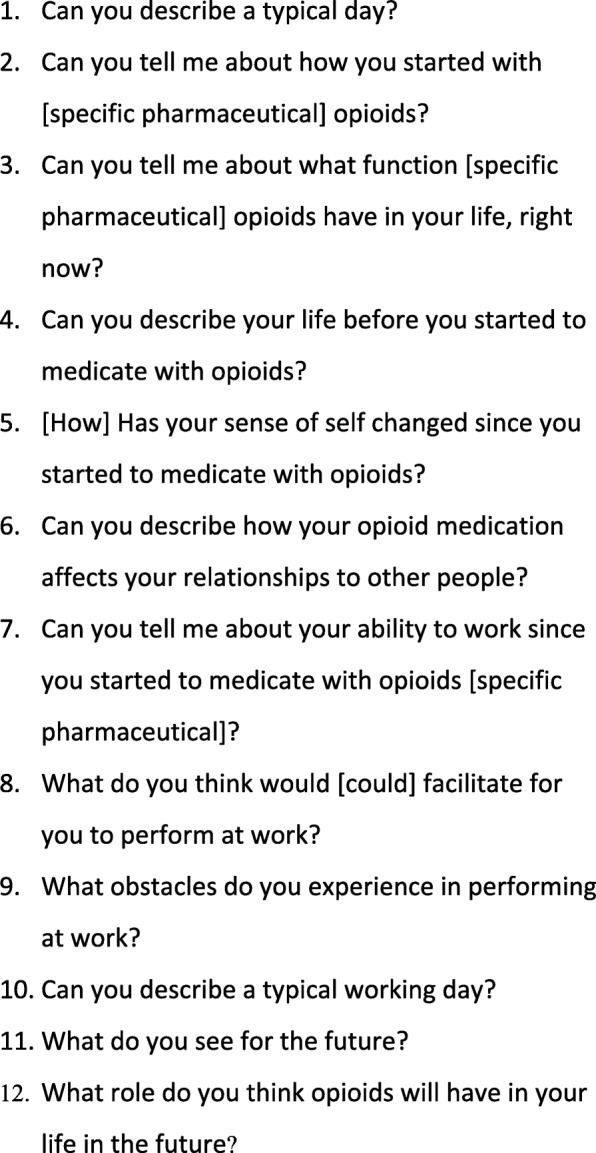
Interview guide used for the semi-structured interviews

The first author (HL) conducted the interviews. HL has long experience as a clinical social worker, working with patients on opioid agonist therapy, and is an experienced clinical interviewer trained in the qualitative method. A semi-structured interview guide based on open-ended questions was used for the interviews and is presented in Fig. 2. The guide was developed with guidance from the literature regarding qualitative methods [31, 33], and important topics were established by going through current literature on research regarding chronic pain and COT. The interview guide was used in a way that could promote openness and allow the informants to discuss the matters most pertinent to them. The participants were encouraged to raise topics of importance to them; by doing so, they led the interview and disclosed their experiences of pain and opioid use. The interviewer was free to probe further on topics generated during the interviews that were consistent with the aims of the study, e.g.: “In what way have you had to change tasks?” “Do you mean…?” The interview guide was piloted once before data collection started, which mainly generated comments on interviewer technique. Interviews lasted between 44 and 75 min. The interviewer had no prior relationship to the participants.

During the interviews with IPA01, IPA02, IPA08, IPA09, and IPA10, an additional researcher participated as an observer (SW or HZ, both trained physiotherapists and PhD students) and was allowed to pose relevant questions.

**Fig. 3:**
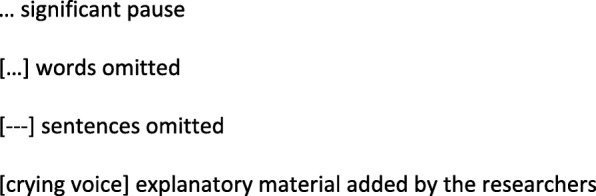
Transcripts notation used in quoted extracts

The interviews were audio recorded and transcribed verbatim for analysis. Transcript notations used in quoted extracts are presented in Fig. 3 in conjunction with the quotes.

*Interpretative phenomenological analysis* (IPA), in accordance with Smith et al. [31], was used for analysing the data. Each transcript was first analysed separately to explore the distinctiveness in each case, as well as experiences, before making more general claims. The transcripts were read and reread several times by the first (HL) and second author (AR). AR is a pain specialist and medical doctor (MD) with long experience of working with patients suffering from chronic pain. She also has extensive experience working with patients with chronic pain and COT, including opioid agonist therapy. The two coders were from different disciplines, to ensure multiple perspectives on pain and opioids in the analysis. The first author followed the process outlined by Smith et al. [31], with a close reading of the transcripts, which generated initial notations that were descriptive, linguistic, and conceptual, to deepen the interpretation of the text. After the initial notations were made, the transcript and notes were entered into the coding program OpenCode 4.02, which is a tool for coding qualitative data generated from text, such as interviews, observations, and field notes [34]. Here, it was used to organise emergent themes and super-ordinate themes closely linked to the transcripts and the initial notes. After each transcript was analysed, the two analysts met to discuss the emergent and super-ordinate themes assigned to the transcript. Through this procedure, themes evolved further and enabled coders to consider the transcripts from different perspectives. This ensured that the interpretations stayed close to the text and the participants’ accounts. When new cases were included in the analysis, efforts were made to bracket, i.e. putting aside, prior ideas and themes that had emerged from previous cases.

After the completion of a separate analysis of every transcript, a cross-case analysis was conducted using the same strategy as for the individual transcripts. Shared themes across cases, relevant to the purpose and aim of this study, were identified, and corresponding texts from the transcripts were assigned accordingly. Patterns and connections between the shared themes were examined, and new super-ordinate themes evolved.

HL’s and AR’s analyses of data were triangulated by the last author (PÅ), who checked for methodological rigor, consistency between themes and quotes, and levels of interpretations. PÅ is a physiotherapist and a full professor in Physiotherapy who is experienced in qualitative method and has clinical expertise in behavioural medicine treatment for persons with chronic pain.

### Methodological rigor

Procedures to enhance the standards of rigor, credibility, auditability, and fittingness were used in this study. These included engaging in reflexivity (e.g., questioning interpretations, becoming aware of one’s own expectations on the data) throughout the research process. The interviewer established the credibility or trustworthiness of the findings [35] by summarising and clarifying ambiguous or indistinct statements during the interviews, and a semi-structured interview guide was used to ensure consistent probing across participants. Further, data were collected through interviews, questionnaires, and medical records. Credibility was also established by using a multi-analyst, interdisciplinary triangulation and through a thorough literature review by the first author. This was done to identify any gaps in the existing literature regarding chronic pain and opioid therapy. To reduce the influence of the literature on the thematic construction, the literature review for comparative analyses with existing research was conducted after the data were analysed. Auditability was established by consistently following the format for coding and sampling, as suggested by Smith et al. [31]. Fittingness, or the transferability of findings, was confirmed by comparative analysis of the findings with existing literature.

## Results

Thirteen persons considered suitable for participation were approached, and 10 agreed to participate. The three individuals that did not participate were all approached by e-mail and did not consent to an appointment for the interview. No reasons were given for choosing not to participate. Recruitment and interviews were conducted between October 2017 and June 2018. Demographics and medical data for the participants are presented in Table 1.

**Table 1:** Demographic data, characteristics and self-reported medical data, (*N* = 10)

	mean (range) or n (%)
Age, years	43 (34–56)
Women	6 (60)
Married/partner	7 (70)
Education level	
Elementary school or less	1 (10)
High school	5 (50)
University	4 (40)
Employment status	
Full time	5 (50)
Part time	1 (10)
Part time with sickness benefits	3 (30)
Unemployed	1 (10)
Pain conditions^a^	
Endometriosis	1 (10)
Fibromyalgia	1 (10)
Postherpetic neuralgia	1 (10)
Arthritis	2 (20)
Inflammatory bowel disease	1 (10)
Headache	1 (10)
Chronic osteomyelitis	1 (10)
Lumbago	5 (50)
Other unspecified pain	1 (10)
More than one pain diagnoses	4 (40)
Duration of pain, years	17 (6–35)
> 10 years	6 (60)
Opioid dose Morphine Milligram Equivalent (MME)^b^	335 (20–960)
> 100 MME/day	8 (80)
Duration of opioid therapy, years 12 (2–31)	
> 10 years	4 (40)

^a^Clinical ICD 10 diagnoses

^b^Opioid doses are reported in approximate morphine milligram equivalent (MME) [36]

Three super-ordinate themes emerged from the analyses: *Without opioids, the pain becomes the boss, Opioids as a salvation and a curse,* and *Acknowledgement of the pain and acceptance of opioid therapy enable transition to a novel self.* The occurrence of super-ordinate themes and themes across individual cases is displayed in Table 2.

**Table 2:** Occurrence of super-ordinate themes and themes across individual cases

Super-ordinate themes and themes	IPA01	IPA02	IPA03	IPA04	IPA05	IPA06	IPA07	IPA08	IPA09	IPA10
Without opioids, the pain becomes the boss	X	X	X	X	X	X	X	X	X	X
The pain separates the self from the lived life	X	X	X	X	X	X	X	X	X	X
The loss of self or the questioned self		X		X	X	X	X	X	X	X
Experience of control and loss of control	X	X	X	X	X	X	X	X	X	X
Opioids as a salvation and a curse	X	X	X	X	X	X	X	X	X	X
A difference between physical dependence and opioid use disorder	X	X	X		X	X	X	X	X	X
Opioids as a menace	X	X	X	X	X	X	X	X	X	X
Acknowledgement of the pain and acceptance of opioid therapy enables transition to a novel self	X	X	X	X	X	X	X	X	X	X
The experience of disbelief and violation – being stigmatised for using opioids	X	X	X	X	X	X	X		X	X
Someone who acknowledges the pain and whom you can trust	X	X	X	X	X	X	X	X	X	X
Acceptance of the pain and opioid therapy as a part of the self	X	X	X	X	X	X	X		X	X

### Without opioids, the pain becomes the boss

The first theme represents how, without opioids, the pain takes control of one’s life which gets invaded and altered. This is pertinent for the understanding of why our participants chose to use opioids, even though they were aware of the potential risks associated with opioid therapy. An illustration of this was provided by IPA08.

Um, and now it feels like, I'll stop taking it [the opioid] now, sure I can quit. That is, I can quit today if that’s how it is, but I know how awful it will be. [---] No, but, one: [with opioids] I’ll be able to live with myself. Two: I’ll be able to live with and have two little children around, who are very demanding, and not lose my patience with them, and I’ll try to also have a life together with my partner or my entire family. Without the pain relief, the pain takes [over]. Absolutely, it is the boss of my life. That’s how it is. **IPA08**

#### The pain separates the self from the lived life

Life before opioid therapy was described as unbearable. The participants described their decision to use opioid therapy as choosing life. To IPA 04, this was like being confined, alienated from life, and dehumanised. The opioids and pain treatment enabled her to participate in life again and regain her sense of self:

Do you know what Teletubbies are, like, the children's series? Before, it was like the pain and the drugs were like this Teletubby suit, and I was somewhere inside trapped behind a zipper [...] it took over everything and there was no human being left. Then, I think that with the help of pain treatment, it is like having the zipper pulled down and stepping out and leaving that suit, which has made me become human. **IPA04**

The opioids gave IPA02 some respite and a feeling of being let out of captivity.

[...] the days when I was able to take medications, it was so nice to get some respite in everyday life in some way, um, from these four white walls; I got out and could do things. [---] So, yes. I could start work again [after taking some opioids], at the same job that I had before, and it was like winning the lottery, I think. After these four years in captivity at home. [---] [laughing] emptying the dishwasher, that was like climbing Mount Everest for me [without opioids]. **IPA02**

#### The loss of self or the questioned self

The lived experience of how chronic pain affected the inner world and the sense of self emerged from the transcripts. The pain was intrusive and made the participants lose their identity and the feeling of control over one’s actions and over external events, here described by IPA04:

Now I am a person with pain; earlier, it was really just a pain problem wandering around [before the opioids] [---] there was SUCH a difference in, like, but this is me again [shaky voice]. **IPA04**

However, some participants feared, or experienced, that the opioids altered their self and their lived experiences of the world. This could be due to how the opioids affected their senses, but also to how others treated them because of their use of opioid medication.

You don’t know what it’s like to be normal anymore, I can sometimes feel like this, but, um, since I’m on such strong medicine. [---] It’s natural to wonder what it would be like without the medicine. **IPA07**

#### Experience of control and loss of control

The chronic pain was experienced as uncontrollable, making life unpredictable and causing a loss of intentionality and agency. Opioids did not take away all of the pain, but made it controllable, thus alleviating the suffering from the disorder and enabled the participants to regain the capacity to act in accordance with how they wanted to live.

The suffering, that now I have pain, now I feel bad, now I don’t want to do anything [−--] [I] would have become more depressed if I knew that this, if this pain would continue, but now I know that I have a medicine that I can take and this pain I have now, it’s only temporary. **[−--]**… my life is under control now actually… with these medications. **IPA06.**

However, it was not only the pain that needed to be under control to enable a wanted life; this applied also to the opioids. The participants used different strategies to accomplish this, e.g., taking medication on schedule, avoiding supplementary doses, or have a limited amount of medication at home. Some participants also tried to take less opioid medication than prescribed, to feel in control of the opioids and the pain:

Yes, it happens, in all honesty, that I try to be without the Ketogan [...] it’s quite rare nowadays, but such experiments have sometimes happened in my life. **IPA04**

Several participants experienced behaviours and priorities that were incongruent with their wanted or true self due to their use of opioids for managing pain. They felt controlled by the drug and at the mercy of their prescribers.

Yes, yes, but you feel, when you are dependent on something, you are like, yes, you are stuck. You must always, if I want to go somewhere, I must make sure not to forget my medicines, eh, if I, uh, have forgotten to renew them, then I have to call and beg and plead. So, you’re constantly dependent on others when you’re dependent on a drug [---] Not having to call and ask, “Oh, please can you give me a refill, I know, oh sorry I, uh, I know I should have called earlier, but this thing happened,” so, oh! **IPA10**

### Opioids as a salvation and a curse

The participants experienced paradoxical effects of the opioids. In spite of the welcome relief from unbearable pain, they experienced stigma from opioid therapy and the problem of being dependent on these drugs, as mentioned above. Thus, from the participants’ accounts, the paradox of opioids as both a salvation and a curse, emerged as an important theme.

#### A difference between physical dependence and opioid addiction

All of the participants were aware of the risk of opioid addiction. By those afflicted it was experienced as a total loss of control.

You get quite resistant to the [opioids] too, or I did, anyway. And I was in such pain too, so I took more and more [...] so I became addicted to them [---] [When] you have pain, then you do anything you can to get rid of that pain, you do anything really; I would probably even buy pills on the street, so to say, so, anything. You do anything. **IPA05**

IPA01 was vigilant of his own behaviour regarding the opioids. He had a previous substance use disorder, mainly of stimulants, and for him it was problematic to use an addictive medicine. He experienced problems with overuse of opioids, which he related to what he called his addictive personality.

Oh, it also happened that I took too much medicine. That I couldn’t, since I had an addiction from before, so it triggers this drug dependency. So I could take all the medication I got for three days so, uh, I could eat all of them the first day. It didn’t work and they were almost forced to intervene [---] It’s a super tough combination of the old demons: so those, the addictive personality, and chronic pain. **IPA01**

For the participants, there was a difference between physical dependence and addiction. As opposed to the examples given above, other participants emphasized that they were not addicted. To them, opioids were not associated with euphoria, or feelings of pleasure, or loss of control. Tolerance was rather seen as a natural consequence of long-term opioid use.

It’s obvious that you get used to it, it is bound to happen, purely medically. So it’s bound to happen… [---] So the receptors have to adapt in some way, nothing else is conceivable. **IPA09**

IPA08, on the other hand, was intimidated by the long-term consequence of opioid use and was vigilant of signs of tolerance or addiction. To her, it was important to know that she could get off the opioids whenever she wanted to.

Um. So, it’s no problem to take it [the opioids] as well. [---] So I would certainly not, so I, I would certainly feel better if I did [increase the opioid dose], but I don’t want to. [---] I'm not going to have a higher dose than this, as well. Nah. [---] You are afraid to get sucked in, or to lose control; I know that it is very easy to do that. **IPA08**

Six of the participants in this study had been offered methadone or buprenorphine agonist therapy due to escalating opioid doses and insufficient pain relief. The switch to methadone or buprenorphine was a turning point for these individuals. Some of them even managed to gradually decrease their opioid doses, once the vicious circle of tolerance, withdrawal, and pain was broken.

It was great when I became, it was [sigh] it was also such an, oh, absolutely fantastic experience when I had gotten the right dose of methadone and experienced being without pain sometimes! It was totally, yeah, crazy! I’d forgotten what it felt like not being, not having pain at all. Feeling painless, sometimes! It was amazing, like my body just soared [laughing]. [−--] now I have more energy when I have gone down from 15 to 10 mg. **IPA10.**

#### Opioids as a menace

All participants had experience of opioids as something problematic because of the association with substance use disorders and addiction. Most of them shared the general view that opioids were problematic and that they should not be easily accessible.

All the doctors know that, most of them know, after all, that it's addictive [---] But I think, is there anyone who really wants to [take opioids]? [---] Nah, but that's not something you want. It is absolutely not something I want. **IPA08**

However, among our participants, opioids were experienced as an effective pain therapy and most of them found it regrettable that the fear of opioids sometimes prevented people from getting an effective pain treatment.

Not everyone understands that it can be a good treatment for pain. But everyone sees the risks everywhere, and it’s obvious that there is a risk. Of course there is. Um, it’s a very potent drug. **IPA09**

### Acknowledgement of the pain and acceptance of opioid therapy enables transition to a novel self

Chronic pain is often an invisible disease, with few pathological findings that can explain the severity of the pain. Many of the participants experienced they had to struggle to convince their care providers of their suffering. However, when the pain and suffering was validated and taken seriously, the participants experienced that they could start to accept the controllable pain and opioid therapy as part of their novel self.

#### The experience of disbelief and violation – being stigmatised for using opioids

For many of the participants, the search for an effective treatment was filled with degrading and violating health care experiences. Several of the participants described how they felt incriminated and considered a nuisance rather than a patient in desperate need of help.

Well, they thought I was [laughing]; they thought I was an addict at the pain clinic in my hometown. Nothing else. That was their starting point. **IPA02**

Being silenced, and not believed or taken seriously could be seen as a denial of help. Here, IPA04 describes a sense of utter despair when she felt accused of malingering:

I mean I’ve been in a hospital bed in the same way and I’ve heard how doctors stood outside the door and said ‘she can’t be in as much pain as she says’ um [shaky voice]. That is incredibly offensive and creates an incredibly powerless despair [crying voice]. I was lying there and could barely get out of the hospital bed because I was in such hellish pain [---]. Yes, it's just silent horror, when I think about it sometimes [crying voice]. **IPA04**

Not being heard or met with compassion led to hopelessness, anger and despair.

Dr X, senior physician: ‘[Participant’s name],’ he said, ‘there is nothing we can do for you, and there are no operations, no medications, nothing.’ So, they refused all pleas for help. Then, I got a letter sent home a few weeks later saying that ‘you are no longer a patient at the pain clinic in the city.’ What? Like that. Well. No one said anything at the meeting. Fucking cowards. [---] And when they say that there’s no chance of it getting better, then I see no chance of ever being able to work again. [---]You shouldn’t say such things to patients at all. You shouldn’t do that… **IPA02**

However, it was not only being denied opioids that could create feelings of abandonment. IPA03 felt that her physician did not engage with her and her treatment when he prescribed opioids as a matter of routine, not paying attention to her escalating doses.

So I can do that, I can think about it, how the hell can you be like that as a doctor? It's simple, ‘I'm prescribing more pills for you so you’ll be quiet for a while’ ... [chuckles] What? **IPA03**

#### Someone who acknowledges the pain and whom you can trust

Not being questioned and doubted made a transition possible and enabled the participants to engage in their treatment and believe in themselves and the possibility to attain their goals once more.

I still see the doctor as an angel in disguise. More like a human angel than a human being as well. Someone who believed in me, someone who had a heart, someone who really wanted to help [crying voice] ... I think that, yes ... it was life-saving. **IPA04.**

When trust was established between the health care providers and the participants, it was easier for the participants to accept suggested treatments.

Because I trusted the doctor. Since she thought that, yes it was the only alternative, the only option [switching to methadone] as the next step, since what I was on then didn’t work. **IPA10.**

It was important to the participants to experience that their care provider understood their predicament, and was knowledgeable about chronic pain *and* opioid treatment.

Yes, exactly, and it was also a bit tough really, um, mentally, because I had no pain doctor and went to the health centre and they just said: “Yes, it’s clear that you have pain, but here are some pills,” kind of. And that was it; they couldn't do anything about the treatment I needed… **IPA09**

#### Acceptance of the pain and opioid therapy as a part of the self

Acceptance of opioid therapy varied between participants. A majority of our participants accepted opioids as a necessity for a tolerable quality of life, while others aimed for a life without opioids. However, abstention from opioids was not the main goal for any of our participants. Instead, they described functional goals as their first priority, e.g., being able to return to work, to function as parents, and to engage in activities important to them, as expressed by IPA09.

And the fact is, as long as it works, it’s fine with me. So, as long as I can live my life, or what should I say. [---] Um, of course, it would have been wonderful to be completely medicine-free. I have eaten tons of medicines of all kinds, but at the same time, and then I had no choice, so to survive and then I chose it because I didn’t want to just lie in bed and be in a lot of pain. **IPA09**

For many of the participants, being weaned off opioids was not seen as be an option since they did not believe that their pain would improve.

I hope that I will be... Painless. From what I understood, I won’t be, but I can try to exercise and I can try to keep it somewhat in check. Together with my medications. My medications and I. **IPA07**

Additional quotations representing the different themes are presented in Table 3.

**Table 3:** Quotations illustrating themes and super-ordinate themes

Super-ordinate themes	Themes	Quotations
Without opioids, the pain becomes the boss	The pain separates the self from the lived life	You would have to say, um, but for me it was, the choice for me was to be disabled, an invalid, or to have a life [with opioids]. [−--] I was in so much pain. Because when I didn’t have the proper pain treatment, then, my body didn’t work, I couldn’t function. I couldn’t sleep, I couldn’t walk; I couldn’t do anything [voice fading]. **IPA09** I played with the notion several times: ‘why should I live when I have this much pain, what’s the point’, kind of, ‘no one believes that I have this much pain and I get no help’ …**IPA05**
	The loss of self or the questioned self	It’s only now really, now that I have tapered the methadone that I, that I realise how much it had affected me mentally [−--] And it is very difficult to admit to yourself that you are affected by it. So, or for me anyway, it is. Em, yeah, I think it’s hard. I haven’t wanted to admit that I am cognitively affected by the opioids. [−--] Yes, but, for example, my sense of humour disappeared. Because you have to think quickly to be witty. **IPA10** Or my belief in people’s attitude, I guess you would say, there is no one around who, I have never experienced that someone has said anything, but it has probably happened. Yes, sometimes knowledge is a burden, you might say, kind of … That you, you know what you are doing and you think, ‘Yes, but I wonder what they believe and what they think and so on.’ **IPA09**
	Experience of control and loss of control	What is really taxing, personally, I think is the chronic pain as well. That you can’t sleep at night, and so that it is more difficult and the opioids work very well on me. **IPA09** That I have a little control too, I take some joy in that I actually manage at this dose… **IPA07** And since I have these old problems with addiction, I don’t dare take a chance either, so I voluntarily come here [to the addiction clinic] 2 times a week and get them. **IPA01**
Opioids as a salvation and a curse	A difference between physical dependence and opioid use disorder	[Oh] I went to the emergency room […] I think I was there three or four times in a week. I just lay there and kind of screamed because I was in so much pain, but then they read in my medical records that I was an addict, so then they didn’t want to prescribe me any painkillers. IPA05 A nurse who doesn’t know my story. Just because I need some extra pain relief, I am treated as if I am some addict, and that’s what I feel bad about, actually. **IPA06** I don’t have withdrawal or that I am addicted or anything like that. I take them because I have pain, that’s how it is, but it’s nothing I feel a craving for. It’s not really something that I crave, it’s not like happy pills, the OxyContin [−--]. Then you’d be an addict. **IPA06** [I] started, I know, with about six [methadone] pills, two pills three times a day. I have actually been able to reduce that dose, so it was like not being able to, yes but kind of lying in bed and screaming because of the pain and not being able to do anything, to have a life again, it made such a difference. **IPA05** Um. So, it’s no problem to take it [the opioids] as well. [−--] So I would certainly not, so I, I would certainly feel better if I did [increase the opioid dose] but I don’t want to. [−--] I’m not going to have a higher dose than this, as well. Nah. [−--] You are afraid to get stuck as well as lose control; I know that it is very easy to do that. **IPA08**
	Opioids as a menace	I think it has to do with this kind of medication, definitively. I wouldn’t tell anybody that I’m om methadone. **IPA07** …I remember that it was discussed a lot – what you… the risks of getting dependent and other risks, ehm, at first it felt more like a problem than a solution to what I was to deal with. **IPA04**
Acknowledgement of the pain and acceptance of opioid therapy enables transition to a novel self	The experience of disbelief and violation – being stigmatised for using opioids	A nurse who doesn’t know my story. Just because I require some extra pain relief, I am treated as if I am some addict, and that’s what I feel bad about, actually. **IPA06** There is nothing for them to do, so very often I believe that they thought I was looking for, um ... [opioid injections] [Sigh]. It feels humiliating and [thoughtfully] ... Yes, but shameful, like, in some way. Nothing that I said or did could make them understand that it wasn’t like that. That feeling of, that people think I am just lying there and [deep sigh] yes, but this whole feeling of powerlessness just came over me. **IPA10**
	Someone who acknowledges the pain and whom you can trust	Oh, I’m afraid I’m not going to get the same doctor, like, that’s my experience if you’re looking for medical care in general today; you certainly can’t expect to meet the same doctor [...] That it could all be done within primary care, just forget about it. **IPA08** The doctor, he believed in me and he told me: ‘It is like this: I will give you this medicine, Oxynorm, but this is what you will get, you will get no extras’, and then he saw that I could manage it…**IPA05**
	Acceptance of the pain and opioid therapy as a part of the self	It can still be hard for me to accept the chronic pain, but it doesn’t take so much energy anymore. I sort of realize that ‘ok, now I’m there again, let’s move on’ **IPA04** It is such a scary disease. I mean it is chronic, I still have it […] It is nothing you get cured from, so it is scary, you never know when the pain will come back, and when it does, then, then… but right now it just feels like I could [−--] Yeah, I hope I will manage without opioids, that would be so cool. **IPA10**

## Discussion

### Summary of the findings

Pain as an intrusive experience was evident in many of the participants’ narratives. As described above, the pain made it hard, sometimes impossible, to live the wanted life and to be as one would like to be and what oneself and others thinks one ought to be [37]. This wanted self was often regarded as the true self, as opposed to the pain-afflicted self. Life with pain was therefore seen as pointless and many of the participants questioned the meaning of life. Opioids could make the pain less intrusive and more predictable, thus controllable, and decreased the discrepancy between the pain-afflicted self and the wanted self. The chronic pain experience as a threat to identity or sense of self is consistent with previous findings [38–40].

### Meaning of the study

The struggle to preserve or regain a sense of self, not affected by either pain or opioids, described by the participants, was similar to what is described as a moral struggle by Edwards et al. [41]. Here, opioids seemed to highlight the moral aspect, for both participants and health care providers. This moral view on opioids might increase the inclination of physicians to focus on either the use of opioids *or* the chronic pain, instead of both [42], thus creating the reductionist approach. The participants experienced this as an under-recognition and under-treatment of pain, and as an inability of health care providers to show compassion for their suffering. This resulted in conflicts and feelings of distrust and abandonment, and sometimes hopelessness. Instead of getting acknowledgement of the complexity of uncontrollable pain and opioid use as a shared problem pertinent for both parties to solve, the participants experienced that their repeated contacts with medical care were seen as drug-seeking behaviours; in particular, the male participants felt accused of being addicts. The participants experienced being stigmatised because of their opioid use and they also felt accused of malingering. Stigma is a common theme in qualitative studies on chronic pain, regardless of opioid therapy [43–45], but seems to be enhanced by the association of opioids with addiction [26–28]. This further increased the discrepancy between the wanted and pain-afflicted self at an inter-personal level.

Our participants’ experiences of opioid dependence and loss of control when trying to manage the pain with opioids fit well with the concept of pseudo-addiction and its three phases [46]. Pseudo-addiction, e.g., drug-seeking behaviour due to poor pain relief [47], is a clinical concept that indicates that under-treatment of pain, rather than craving for the drug, is what drives the patient’s problematic opioid use. This is, however, not an empirically verified concept; furthermore, it has been questioned, not least due to the opioid epidemic in the U.S. and the related over-prescription of opioids [48]. The first phase is the *onset of pain,* when the patient receives inadequate analgesia and requests more medication, often preferring a specific drug, which raises suspicion among health care providers. The next phase is the *escalating phase*, where the patient has to convince the care provider of the legitimacy of their pain and, by extension, their opioid use. This was apparent when the participants stated that they had to “earn” their opioids, sometimes by impersonating a trustworthy patient by accepting other treatments, or displaying expected behaviours. In the *final phase, the crisis*, the unrelieved pain continues, the patient engages in increasing and sometimes bizarre drug-seeking behaviours, leading to mistrust, anger, and isolation on the part of the patient, and frustration and avoidance on the part of the health care providers [46]. This was evident in many of our participants’ narratives as experiences of disbelief and violation. For some of the participants who experienced escalating opioid doses, a switch to methadone or buprenorphine, used in opioid maintenance and agonist therapy, was used to regain control and end the downward spiral of chronic pain and escalating opioid use. This is consistent with opioid agonist treatment with methadone or buprenorphine as a highly efficient treatment of opioid addiction [49, 50]. Further, the similarities and the synergistic effects of opioid dependence and chronic pain are well-known, where one condition might exacerbate the other [3, 51].

However, it was not only the opioid medication that led to a transition for the participants. Being met with compassion and having their suffering acknowledged and validated engendered feelings of hope, which seemed to enable the participants to renegotiate their previous self, thus defining a novel version of self and life. Validation can be described as communication of empathy, acceptance, and understanding; it has a positive impact on adherence to treatment and is known to help in regulating negative emotions [52]. When patients experience validation and compassion, they often feel more satisfied; furthermore, clinicians who manage to validate their patients and show compassion for their predicaments are often able to give the patients what they have long sought – a feeling of being cared for [53, 54] – and decrease the discrepancy between the wanted and the pain-afflicted self at an inter-personal level [54].

Perhaps this is what Gadamer meant when he claimed that a physician has a twofold obligation: to unify his/her specialised knowledge with a partnership, that is, to try to comprehend the first person (patient) experience, while deciding what treatment to offer [55] [p. 35, Swedish edition]. Our participants experienced this as making amends, helping them to regain trust in health care and themselves. Thus, it became easier to accept the suggested treatment alternatives and find acceptance for the tolerable pain (with opioids) as part of life and a novel self.

### Methodological considerations

As previously described, several steps were taken to strengthen the trustworthiness of the findings. Nevertheless, the findings need to be interpreted with some limitations in mind. Recruitment of participants went through the treating physicians, entailing a risk that participants were chosen for other qualities than being suitable for this study, e.g., a good relationship with the physician or good treatment response. Nonetheless, all our participants had long histories of opioid therapy, and a variety of experiences related to pain and opioid treatment, generating data with richness and depth. This was regardless whether only the interviewer or both interviewer and observer were present. Further, the findings are based on a small selected group of individuals with chronic pain and ability to work, treated in tertiary care. In accordance with the IPA methodology, with detailed accounts of individual experiences, empirical generalizability is not sought; rather a theoretical transferability, where the reader can make links between the findings, the extant literature, and their own professional experience [31].

### Clinical implications

Interdisciplinary multimodal treatment is an evidence-based treatment for complex chronic pain [56, 57]. Chronic opioid therapy could be part of such treatment although its evidence is weak [17]. Our study illustrates that specifics of a particular case, such as opioid sensitivity and measurable improvement of function and quality of life, could justify the long-term use of opioids. This requires consideration and monitoring of adverse events such as tolerance, problematic use, and other side effects, due to the limitations and dangers of high-dose long-term opioid therapy. If problems should arise for the patients who already use opioids, the solution cannot lie in abandoning or rejecting them, as they struggle with their pain, and sometimes, also with established dependence. Managing pain with opioids therefore requires a close cooperation and a trusting relationship between the patient and the responsible physician, where both benefits and risks with COT can be discussed and assessed. In a clinical setting, this means informing patients about the mechanism of opioid dependence and opioids sometimes paradoxical effects on chronic pain. It also requires continuous updating of treatment goals, taking into account the patient’s experiences of living with chronic pain and how the opioid therapy assists in reaching these goals. This includes making the life experience meaningful.

This study also underlines the stigma related to chronic pain, and especially chronic pain and opioids. It highlights the importance of an ethical awareness on the part of the health care professionals, treating an already vulnerable group.

## Conclusion

Participants used opioids as a way to regain control over their pain, thus reclaiming the wanted life and self, and a sense of control over one’s life-world. However, using opioids to manage pain was not unproblematic; some of the participants had experienced a downward spiral of escalating pain and uncontrollable opioid use, resulting in tolerance, withdrawal, and drug-seeking behaviours. In line with previous phenomenological research, participants emphasised the importance of control, regarding both predictions of pain and opioid use, irrespective of whether they had experienced problematic opioid use or not. They had also experienced being stigmatised because of their opioid use. Therefore, it was important for the participants that trust was established between them and their health care providers. Regardless of the potential sociocultural benefits of employment, the participants had experiences of balancing positive and negative effects of opioid therapy, similar to what previous qualitative research has found.

## Data Availability

The transcripts and qualitative data matrix used and/or analysed during the current study are available from the corresponding author on reasonable request.

## Abbreviations

COT: chronic opioid therapy
IPA: Interpretative phenomenological analysis
